# Deprived areas and community water fluoridation in Brazil: a multilevel approach for refocusing public policy

**DOI:** 10.4178/epih.e2021031

**Published:** 2021-05-01

**Authors:** Franklin Barbosa da Silva, José Leopoldo Ferreira Antunes, Paulo Frazão

**Affiliations:** 1 Public Health Graduate Program, University of São Paulo, São Paulo, Brazil; 2 Department of Epidemiology, Public Health School, University of São Paulo, São Paulo, Brazil; 3 Department of Politics, Management and Health, Public Health School, University of São Paulo, São Paulo, Brazil

**Keywords:** Fluoridation, Drinking water, Sanitation, Public policy, Health policy, Multilevel analysis

## Abstract

**OBJECTIVES:**

The aim of this study was to determine whether geographic location, socioeconomic status, infant mortality, and mortality from diarrheal disease in health regions are associated with the provision of community water fluoridation (CWF) in Brazilian municipalities.

**METHODS:**

A multilevel ecological study was conducted based on data from the National Survey of Basic Sanitation and Human Development Atlas. A multilevel analysis was carried out considering Brazilian municipalities as the first level and health regions as the second level, comprising sanitation, demographic, socioeconomic, and health characteristics.

**RESULTS:**

The observation units comprised 5,565 municipalities clustered in 438 health regions in Brazil. The lack of CWF provision was positively associated with the following municipal characteristics: a below-median proportion of inhabitants covered by the sewage network, medium to very low human development index, below-median per capita gross domestic product, and an above-median percentage of expenditures on sanitation. In relation to the health regions, the likelihood of a lack of CWF provision was greater in the municipalities belonging to the health regions located in the Northern and Northeastern areas of Brazil and in those where child mortality due to acute diarrheal disease and the proportion of people with low income were higher when adjusted by municipal indicators.

**CONCLUSIONS:**

Information on the characteristics associated with CWF provision constitutes important input for refocusing public policy to reduce inequalities among Brazilian municipalities and health regions. These findings may help policy-makers to understand the challenges facing CWF expansion in low-, middle-, and high-income countries.

## INTRODUCTION

Environmental conditions, most notably sanitation conditions, are a noteworthy dimension of development and one of the most significant determinants of health [1]. Untreated tooth decay is the most prevalent disease worldwide and strategies with recognized effectiveness for its control at the population level, such as fluoride-adjusted water and toothpaste, should be prioritized [2].

Despite the importance of such measures, inequalities in coverage among federative units can still be found in countries that provide water fluoridation, such as among states and territories in Canada, the United States, and Australia, and among municipalities in Brazil [3]. The level of human development and population size are connected with the provision of water fluoridation [1,4]. The provision of community water fluoridation (CWF) is also related to the coverage of sanitation services, provider characteristics, the percentage of the urban population, and the geographic location of the municipality in the country [5,6].

When health events are investigated, specific risk factors are usually evaluated separately. However, when more comprehensive investigations are carried out, it may be possible to observe close relationships between the environment and the outcomes studied [7]. Thus, certain socioeconomic and health conditions that characterize the territorial context of municipalities could be associated with the implementation of public health strategies, such as CWF. Territories with high social vulnerability are characterized by high child mortality rates, a high prevalence of diarrheic diseases, and a high proportion of low-income people.

Mortality caused by diarrheic diseases is closely related to sanitation and environmental conditions as a whole [8,9], which, in turn, depend on the quality of hydric resources [10]. Inadequate drinking water, sanitation (sewer connections), and hygiene (handwashing) are important risk factors for diarrheic diseases [11]. From 2000 to 2015, 8.5% of deaths of under-5 children were caused by diarrheic diseases [12]. The United Nations defined in its agenda for sustainable development that, by 2030, all avoidable deaths of newborns and under-5 children should no longer occur; and that all countries should reduce total mortality in those age groups to less than 25 per 1,000 live births [13]. High poverty areas usually have precarious sanitation and vice-versa [11,14].

Sanitation conditions, family income, and access to public health strategies are often associated with child mortality; therefore, immunization, control of infectious diseases, and access to treated water are considered effective control measures [15,16]. In this sense, scientific information on the association between territorial characteristics related to the federative units of a country and the provision of public health strategies (for instance, CWF) could be extremely helpful as a way to provide new directions for public policies. Considering the lack of knowledge about the implications of territorial conditions on CWF provision, the present study aimed to determine whether geographic location, socioeconomic conditions, child mortality, and mortality due to diarrheic diseases were associated with the lack of CWF provision in Brazilian municipalities analyzed according to health regions.

## MATERIALS AND METHODS

We undertook a cross-sectional multilevel ecological study involving all Brazilian municipalities based on information from official agencies. Brazilian municipalities were distributed into 438 health regions, organized according to inter-municipal arrangements determined at the state level, aimed at integrating the organization, planning, and execution of health actions and services [17]. Regionalization is a strategy of the health sector that, among other aspects, is aimed at improving the response capacity and degree of accountability concerning the health needs of the population [18].

The variables at the first and second levels were selected in order to verify the possible associations between characteristics of the health regions and the provision of fluoridation by municipalities.

### Data sources

The 2010 Demographic Census contained information on the characteristics of households and their residents. The National Research on Basic Sanitation (*Pesquisa Nacional de Saneamento Básico – PNSB*) provided data on basic sanitation conditions in 2008 for all Brazilian municipalities [19]. The Atlas of Human Development had indicators for all Brazilian municipalities and states [20]. The web portal of the Informatics Department of Brazilian Health System (*DATASUS*) offered data on life statistics (mortality and live births), morbidity, incapacity, access to health services, procedures of healthcare, living conditions and environmental factors [21]. The web portal of the Secretariat of the National Treasury provided accounting and fiscal information of states, municipalities, and the Federal District [22].

### Outcome variable

The outcome was defined as the lack of CWF provision in the municipality obtained from the *PNSB*.

### Characteristics of the municipalities (level 1)

The variables related to the municipalities were the percentage of households served by water distribution system and by sewerage and wastewater collection systems (data were provided by the 2010 Demographic Census, and the median values of both percent values were used); population size (estimates for 2010 were used and separated into 3 categories—< 10,000, 10,000-50,000, and > 50,000—according other studies [4]); the percentage of the population living in the urban area of the municipality (provided by the 2010 Demographic Census; the quartiles of the distribution were calculated and the municipalities were grouped into 2 categories: urban population < 47 and ≥ 47%); the gross domestic product (GDP) per capita defined by dividing the GDP of the municipality by the number of inhabitants (the values were presented in Brazilian reais, using values from 2010 for reference, and the median values were used to categorize GDP as < 12,867 and ≥ 12,867 reais); the percentage of expenditures spent on sanitation in the municipality from 2005 to 2008, defined by dividing those expenses by the total expenses spent on all sectors and multiplying the result by 100 (data were provided by Secretariat of the National Treasury and the median value was used to categorize the variable); and the municipal human development index (MHDI), which is a measure composed of 3 dimensions (longevity, education, and income) that has values ranging from 0 to 1, with values closer to 1 indicating higher the human development (using 2010 data, 3 categories were defined: very low and low [< 0.599]; medium [0.600-0.699]; and high and very high [≥ 0.700]).

### Characteristics of the health regions (level 2)

The Brazilian health system has recognized the importance of contextual factors for health events, and the health regions identified by municipal and state managers, based on cultural, economic, and social identities, communication networks, and shared transportation infrastructure are the expression of the institutional efforts in this direction [23]. Considering the conditions that characterize territories with high social vulnerability, the following variables provided by *DATASUS* for each health region were selected: the proportion of low-income people (defined by the proportion of people whose income was lower than 50% of the minimum wage, using values from 2010, when that value was $510.00 Brazilian reais); the infant mortality rate (defined by the number of deaths of those under the age of 1 per 1,000 live births); and the mortality rate due to acute diarrheic disease (ADD), defined as the percentage of under-5 children deaths caused by ADD according to the codes from A01 to A09 from International Statistical Classification of Diseases and Related Health Problems, 10th revision. All variable values were grouped into tertiles for the analysis.

The geographic locations of the health regions were analyzed. Health regions were distributed across the 5 main Brazilian geographic divisions (North, Northeast, Southeast, South, and Midwest); this classification has been used in studies related to the provision of sanitation services [24]. A previous study showed uneven coverage of CWF between the South/Southeast and North/Northeast macro-regions [6,25], a trajectory that seems to follow the history of the implementation of other essential public services, such as access to the general water system and the electric energy system [26].

### Statistical analysis

At first, the lack of CWF provision was evaluated in the presence of each variable at both levels (municipality and health regions). Next, we calculated the prevalence ratios (PRs) and respective 95% confidence intervals (CIs) using Poisson regression with robust variance. A multiple analysis was then carried out, including all level 1 variables, aimed at verifying which municipal characteristics would remain associated in the presence of all the others. The other models were constructed to check whether characteristics connected with the health regions would affect the outcome, and to assess the effect of first level variables on the outcome. For that purpose, a multilevel Poisson regression analysis was carried out using a random-effects model. Level 1 variables were included in all models, and for each multilevel model a level 2 variable was aggregated, thus resulting in a total of 4 models. The -2 log-likelihood statistic was used to evaluate the adjustment quality of the models. Stata version 12 (StataCorp., College Station, TX, USA) was used.

### Ethics statement

The research used data from census and other official sources, of public access. According to the Resolution of the National Health Council (CNS) No. 510, of April 7, 2016, no mandatory evaluation by the national system of research ethics committees is needed.

## RESULTS

Out of the 5,565 Brazilian municipalities, 60% had water fluoridation services. Data on the water distribution system (n= 5) and sewerage and wastewater collecting system (n = 111) were not available for a few municipalities. There were 438 health regions, and information on the variables gathered at this level was not lacking. Table 1 presents the distribution of the outcome among variable categories at both levels. A lack of CWF was more frequent in municipalities with values lower than the medians of the sanitation variables, as well as in municipalities with fewer than 10,000 inhabitants and in those where the MHDI was low/very low. A lower percentage of urban area and a lower GDP of the municipality were associated with a lower likelihood of CWF. An inverse correlation was found as to the percentage of sanitation investments: municipalities with higher proportional investments in the sector presented a lower percentage of public policy provision. In relation to the characteristics of the health regions (second-level variables), we observed that the higher tertiles of infant mortality and mortality caused by ADD were associated with a lower presence of CWF in the corresponding health regions. The higher the proportion of low-income people living in the health region, the most remarkable was the absence of CWF services. In the health regions in the North macro-region, the absence of CWF was 90.0% higher than in the other macro-regions.

Table 2 displays the values of the simple analysis for each variable in the first column, and the adjusted values including only level 1 variables in the second column. It is worth noting that all variable categories were associated with the outcome, except for second-level variables for the Southeast macro-region (PR, 1.02; 95% CI, 0.95 to 1.09), which showed no significant difference in comparison to the South macro-region, which was used as the reference. The results demonstrated plausibility for the construction of the other models. As shown in the second column, the percentage of households served by a water distribution system, population size, and the percentage of the population living in urban areas did not maintain an association with the outcome. The low/very low category of the 2010 MHDI was associated with a lack of CWF (PR, 1.29; 95% CI, 1.19 to 1.40), followed by the medium category (PR, 1.17; 95% CI, 1.10 to 1.25). When compared to areas with higher-than-median values, those with a lower-than-median GDP per capita showed 16% higher odds of not providing CWF.

Table 3 displays the multilevel analysis models constructed to test the hypothesis of the study. Model 2 included the mortality rate by ADD and model 3 included the proportion of low-income people. Concerning these characteristics connected with the health regions, the first-level variables behaved quite similarly to what was observed in the previous model, with the same variables categories maintaining associations. The only difference was found in second-level categories, where only lower tertiles of the mortality rate by ADD (PR, 1.07, 95% CI, 1.01 to 1.14) and the proportion of low-income persons (PR, 1.15; 95% CI, 1.05 to 1.25) did not lose significance. Model 4 included the infant mortality rate. Higher probabilities of the outcome were associated with the low/very low category of the MHDI (PR, 1.22; 95% CI, 1.12 to 1.33) and lower-than-median values of GDP per capita (PR, 1.15; 95% CI, 1.09 to 1.23). As to the mortality rate, both lower tertiles maintained the association. In the fifth model, where besides municipalities’ characteristics, geographic location was added, the associations were maintained for municipalities with fewer than 10,000 inhabitants (PR, 1.11; 95% CI, 1.02 to 1.21) and above-median percentages of expenditures spent on sanitation (PR, 1.08; 95% CI, 1.02 to 1.13). The PRs for the geographic location categories remained close to the values observed in the simple analysis. A high probability of not having CWF services was found in the health regions located in the Northeast and North macro-regions (PR, 1.46; 95% CI, 1.33 to 1.61 and PR, 1.63; 95% CI, 1.47 to 1.82, respectively). It must be noted that, in all models, the percentage of households served by a water distribution system lost its association with the outcome, while municipal expenditures on sanitation remained associated.

## DISCUSSION

This study evaluated the effects of from regional characteristics, adjusted by factors associated with municipalities, on the lack of CWF provision. The associations between the outcomes and municipal characteristics reported in other studies were confirmed [3-5]. However, after the adjustment enabled by model 1, it was found that lower coverage of sewerage and wastewater collecting systems, lower human development, lower GDP per capita, and a higher percentage of expenditures spent on sanitation were strongly associated with a lack of CWF. The main hypothesis of the study was confirmed—that is, regardless of municipality-level factors, the absence of CWF provision was associated with territories that have been historically neglected and have high social vulnerability, as measured by high levels of poverty, child mortality, and diarrheic disease.

The results confirmed the association between municipal characteristics and the outcome. In the adjusted analysis, some differences were found when compared with reports in the literature. Researchers have suggested that remarkable inequalities in CWF could be associated with the exclusion of rural districts [6]. In the present investigation, this relationship was not consistent when considering other factors related to the municipality. Among a number of social and economic indicators at the municipal level investigated in the state of Santa Catarina, Brazil, only the population size of the municipalities maintained a significant association with CWF provision in the presence of other factors [3], a result that does not match what was found in this study, where population size was not associated with the outcome in almost all adjusted models. This discrepancy may be imputed to the fact that this study analyzed information from all municipalities of the country while the previous study [3] was restricted to a single state in the South macro-region. A study comprising all municipalities in the country found more widespread fluoridation provision in more populous municipalities with a higher human development level [4]. In the present study, the human development level remained associated with the outcome, regardless of other municipality-level factors.

A study involving all Brazilian municipalities pointed out a possible relationship between the quality of sanitation services provided by companies and CWF. Municipalities with lower coverage of water and sewerage systems presented the lowest rates of CWF provision [5]. This information was refined in this research. In the presence of other variables, the percentage of households served by a water distribution system lost its importance, most likely due to the high coverage rates (very close to universal in most municipalities). The findings confirmed the relevance of socioeconomic factors and those involving the quality of the sanitation services in municipalities, particularly the sewerage and wastewater collecting systems, for the outcome.

Among municipal variables, the only item that maintained an association with the outcome in all models was expenses spent on sanitation, showing that higher municipal investment was associated with a lower probability of provision of CWF. A possible explanation is that, in municipalities with higher expenses, the sanitation system may be in a state of structural expansion, thus requiring larger investments into that sector by the local management. Data on the need for investments per capita to provide universal sanitation services in Brazil until 2025 showed that the Southeast macro-region would need half of all investments when compared with the Northeast macro-region, which is precisely the one with the worst infrastructure in the country [27].

The practice of adjusting the natural concentration of fluoride in water treatment plants to improve dental health depends on multiple factors, including the quality of the sanitation services rendered, which may vary according to the characteristics of hydric resources in the territory. Sanitation is a complex intervention that might exhibit dynamic interactions between psychologic-behavioral determinants and environmental, social, cultural, political, and economic parameters that might operate at different scales [28]. The present study confirmed that territorial characteristics connected to the health regions were associated with the lack of CWF provision.

Infant mortality rates and deaths due to diarrheic disease are indicators associated with the provision of sanitation services [16,29]. In the present study, higher mortality rates were associated with a lack of CWF provision. This result may have immediate practical implications for public policy coordination. As these are basic health indicators, reliable data in the regional context are usually available, thus making it easier to recognize localities likely to lack fluoridation and where CWF implementation must be prioritized. Moreover, because public policy is connected with sanitation conditions, the indicators may suggest a need for planning and regulation activities in the sector, thus leading to the inclusion of CWF into the political agenda.

The absence of CWF was associated with municipalities with lower GDP per capita and regions with higher poverty levels (model 3). Researchers found that no population groups were exposed to adequate fluoride in drinking water (natural or artificial) in low-income countries, whereas the corresponding proportion was 20.9% in high-income countries. If it is accessible for the entire population, CWF might reduce socioeconomic inequalities in dental caries [30]. Therefore, the existence of a system or structure for water treatment and distribution reaching both rich and poor areas is an essential condition for CWF as a public policy. There is no other way for CWF to be an immediate protective factor for all domiciles connected to the system, regardless of the socioeconomic condition of each family [4]. At a country scale, as shown in this study, there is remarkable inequity in CWF provision, suggesting the need to adjust implementation strategies including an inter-federative coordination of public policy. Otherwise, federative countries with 3 levels of government with considerable autonomy and interdependence, as is the case for Brazil, would face difficulties in reaching the results that would be expected from a measure with strong potential for promoting equity. In Brazil, a major reason for the inequality in CWF provision is related to the sanitation model adopted in the country: the distribution and access to fluoridation services were not rooted in the principle of equity, but were instead predominantly based on a strong entrepreneurial conception, whereby investments were driven by public works that would lead to high profitability and rapid return of the capital invested [4].

Municipality-level characteristics were not consistently associated with the outcome when the macro-regions were considered (model 5), showing that the magnitude of the association increases moving from the South macro-region municipalities towards those in the North macro-region. This finding seems to reflect the tremendous territorial inequalities that traditionally marked— and continue to do so—the social and economic development of Brazil, producing disparities among Brazilian citizens in education, health, and income levels. A comprehensive 5-decade study showed that inequalities have been markedly reduced over recent decades, although Brazil remains one of the most unequal countries in the entire planet. Access to public goods, such as clean water, sanitary drainage, and electrical power, followed a trajectory where increasing coverage went from the South and Southeast macro-regions towards the Midwest, Northeast and North macro-regions [26]. In the present study, the lack of CWF provision was connected with that trajectory, resulting from a pattern of social and economic development that has historically neglected certain territories inside the country [26]. Oral health data from 2003 and 2010, the 2 largest and earliest surveys in the country, showed a higher frequency of dental caries for 12-year-old children [31] and adults [32] and a lower frequency of dental fluorosis [33] in the Midwest, Northeast, and North macro-regions than in the South and Southeast macro-regions, where municipalities had better socioeconomic conditions and CWF provision.

Although the data used in the present study were obtained in 2008 and 2010, the study analyzed phenomena where changes take place over the medium to long term. Information on the CWF situation was obtained by means of a questionnaire provided by the main Brazilian official agency for geography and statistics, and entities that rendered sanitation services; thus, these were the best and most comprehensive data sources available for CWF in Brazil. A lack of data for some variables was observed in a small number of municipalities and would not change the associations observed. Despite the analytical importance of the study, due to its cross-sectional nature, no causality can be inferred between the characteristics analyzed and the outcome.

The findings showed that a lack of CWF provision was associated with municipal characteristics related to the sewage network, human development level, GDP per capita, and expenditure on sanitation. Adjusted by municipal indicators, territorial characteristics of health regions related to infant mortality, ADD, and the proportion of people with low income were also associated with a lack of CWF. Information on the characteristics associated with CWF provision in the municipalities is relevant for a re-orientation of public policies to reduce inequalities among Brazilian regions and between municipalities concerning CWF implementation. These findings may help policy-makers to understand the challenges facing CWF expansion in low-, middle-, and high-income countries.

## Figures and Tables

**Table 1.** Distribution of sanitation, demographic, socioeconomic, and health characteristics across municipalities and health regions, showing the absolute and relative frequency of municipalities that did not provide community water fluoridation for each variable in Brazil, 2008

Characteristics	n	Non-fluoridated, n (%)
Town-level		
Households served by potable water supply (median %)		
≥72	2,780	765 (27.5)
<72	2,780	1,444 (51.9)
Households served by sewage collection system (median %)		
≥17	2,727	654 (24.0)
<17	2,727	1,484 (54.4)
Population size		
>50,000	608	156 (25.7)
10,000-50,000	2,444	1,003 (41.0)
<10,000	2,513	1,055 (42.0)
Human development level - 2010		
Very high and high (≥0.700)	1,933	240 (12.4)
Medium (0.600-0.699)	2,233	985 (44.1)
Low and very low (<0.599)	1,399	989 (70.7)
Urban population proportion (median %)		
≥47	4,174	1,446 (34.6)
<47	1,391	768 (55.2)
Per capita gross domestic product (median)		
≥12,867	2,782	516 (18.5)
<12,867	2,783	1,698 (61.0)
Budget percentage of sanitation expenditures (median %)		
<0.66	2,787	1,002 (35.9)
≥0.66	2,778	1,212 (43.6)
Health regions		
Deaths due to acute diarrheal disease in under-5 children (tertiles %)		
<2.22	146	336 (18.0)
2.22-4.88	146	630 (34.1)
>4.88	146	1,248 (67.5)
Individuals with income below half the minimum wage (tertiles %)		
<25.49	146	248 (13.3)
25.49-55.45	146	735 (39.5)
>55.45	146	1,231 (67.0)
Infant mortality rate - deaths per thousand (tertiles)		
<16.46	146	348 (18.7)
16.46-20.48	146	814 (44.0)
>20.48	146	1,052 (56.9)
Geographic macro-region		
South	68	145 (12.2)
Southeast	153	235 (14.1)
Midwest	39	212 (45.5)
Northeast	133	1,218 (69.9)
North	45	404 (90.0)

**Table 2.** Crude and adjusted values for associations between lack of water fluoridation provision and municipality and health region-level characteristics in Brazil, 2008

Characteristics	Crude values	Model 1
Town-level		
Households served by potable water supply (median %)		
≥72	1.00 (reference)	1.00 (reference)
<72	1.17 (1.11, 1.22)^***^	1.04 (0.98, 1.09)
Households served by sewage collection system (median %)		
≥17	1.00 (reference)	1.00 (reference)
<17	1.23 (1.17, 1.29)^***^	1.10 (1.05, 1.16)^***^
Population size		
>50,000	1.00 (reference)	1.00 (reference)
10,000-50,000	1.11 (1.02, 1.20)^*^	0.97 (0.89, 1.05)
<10,000	1.13 (1.04, 1.23)^**^	1.00 (0.92, 1.08)
Human development level - 2010		
Very high and high (≥0.700)	1.00 (reference)	1.00 (reference)
Medium (0.600-0.699)	1.28 (1.20, 1.35)^***^	1.17 (1.10, 1.25)^***^
Low and very low (<0.599)	1.50 (1.41, 1.60)^***^	1.29 (1.19, 1.40)^***^
Urban population proportion (median %)		
≥47	1.00 (reference)	1.00 (reference)
<47	1.13 (1.08, 1.20)^***^	0.97 (0.92, 1.03)
Per capita gross domestic product (median)		
≥12,867	1.00 (reference)	1.00 (reference)
<12,867	1.35 (1.28, 1.41)^***^	1.16 (1.09, 1.23)^***^
Budget percentage of sanitation expenditures (median %)		
<0.66	1.00 (reference)	1.00 (reference)
≥0.66	1.05 (1.00, 1.10)^*^	1.07 (1.02, 1.12)^**^
Health regions		
Deaths due to acute diarrheal disease in under-5 children (tertiles %)		
<2.22	1.00 (reference)	-
2.2-4.88	1.18 (1.10, 1.25)^***^	-
>4.88	1.28 (1.20, 1.36)^***^	-
Individuals with income below half the minimum wage (tertiles %)		
<25.49	1.00 (reference)	-
25.49-55.45	1.20 (1.13, 1.28)^***^	-
>55.45	1.46 (1.38, 1.55)^***^	-
Infant mortality rate - deaths per thousand (tertiles)		
<16.46	1.00 (reference)	-
16.46-20.48	1.20 (1.13, 1.28)^***^	-
>20.48	1.32 (1.24, 1.41)^***^	-
Geographic macro-region		
South	1.00 (reference)	-
Southeast	1.02 (0.95, 1.09)	-
Midwest	1.30 (1.18, 1.42)^***^	-
Northeast	1.50 (1.40, 1.59)^***^	-
North	1.70 (1.55, 1.84)^***^	-
-2 log-likelihood	Null model	Model 1
	13,365.26	12,855.80

Values are presented as prevalence ratio (95% confidence interval).

* p<0.05,

** p<0.01,

*** p<0.001.

**Table 3.** Adjusted values of associations between the lack of water fluoridation provision and municipality and health region-level characteristics in Brazil, 2008

Characteristics	Model 2	Model 3	Model 4	Model 5
Town-level				
Coverage of potable water supply (median %)				
≥72	1.00 (reference)	1.00 (reference)	1.00 (reference)	1.00 (reference)
<72	1.04 (0.99, 1.09)	1.04 (0.99, 1.10)	1.04 (0.98, 1.09)	1.04 (0.99, 1.10)
Coverage of sewage collection system (median %)				
≥17	1.00 (reference)	1.00 (reference)	1.00 (reference)	1.00 (reference)
<17	1.10 (1.04, 1.16)^***^	1.10 (1.04, 1.16)^***^	1.11 (1.05, 1.17)^***^	1.04 (0.98, 1.11)
Population size				
>50,000	1.00 (reference)	1.00 (reference)	1.00 (reference)	1.00 (reference)
10,000-50,000	0.97 (0.90, 1.04)	0.98 (0.90, 1.06)	0.97 (0.90, 1.06)	1.03 (0.95, 1.12)
<10,000	1.00 (0.92, 1.08)	1.02 (0.94, 1.11)	1.01 (0.93, 1.09)	1.11 (1.02, 1.21)^*^
Human development level - 2010				
Very high and high (≥0.700)	1.00 (reference)	1.00 (reference)	1.00 (reference)	1.00 (reference)
Medium (0.600-0.699)	1.17 (1.10, 1.24)^***^	1.11 (1.04, 1.20)^**^	1.12 (1.05, 1.20)^**^	1.06 (0.99, 1.13)
Low and very low (<0.599)	1.27 (1.17, 1.38)^***^	1.18 (1.07, 1.30)^**^	1.22 (1.12, 1.33)^***^	1.05 (0.95, 1.16)
Urban population rate (median %)				
≥47	1.00 (reference)	1.00 (reference)	1.00 (reference)	1.00 (reference)
<47	0.97 (0.92, 1.03)	0.97 (0.92, 1.03)	0.98 (0.92, 1.03)	1.02 (0.96, 1.08)
Per capita gross domestic product –Brazilian reais (median)				
≥12,867	1.00 (reference)	1.00 (reference)	1.00 (reference)	1.00 (reference)
<12,867	1.14 (1.08, 1.22)^***^	1.13 (1.06, 1.20)^***^	1.15 (1.09, 1.23)^***^	1.00 (0.93, 1.08)
Budget percentage of sanitation expenditures (median %)				
<0.66	1.00 (reference)	1.00 (reference)	1.00 (reference)	1.00 (reference)
≥0.66	1.07 (1.02, 1.12)^**^	1.06 (1.01, 1.11)^**^	1.07 (1.02, 1.12)^**^	1.08 (1.02, 1.13)^**^
Health regions				
Deaths due to acute diarrheal disease in under-5 children (tertiles %)				
<2.22	1.00 (reference)	-	-	-
2.22-4.88	1.06 (1.00, 1.12)	-	-	-
>4.88	1.07 (1.01, 1.14)^**^	-	-	-
Individuals with income below half the minimum wage (tertiles %)				
<25.49	-	1.00 (reference)	-	-
25.49-55.45	-	1.06 (1.00, 1.14)	-	-
>55.45	-	1.15 (1.05, 1.25)^**^	-	-
Infant mortality rate - deaths per thousand (tertiles)				
<16.46	-	-	1.00 (reference)	-
16.46-20.48	-	-	1.11 (1.04, 1.17)^***^	-
>20.48	-	-	1.13 (1.05, 1.20)^**^	-
Geographic macro-region				
South	-	-	-	1.00 (reference)
Southeast	-	-	-	1.04 (0.96, 1.13)
Midwest	-	-	-	1.28 (1.16, 1.41)^***^
Northeast	-	-	-	1.46 (1.33, 1.61)^***^
North	-	-	-	1.63 (1.47, 1.82)^***^
-2 log-likelihood	12,849.208	12,844.587	12,840.729	12,760.065

Values are presented as prevalence ratio (95% confidence interval).

* p<0.05,

** p<0.01,

*** p<0.001.

## References

[1] Frazão P, Narvai PC (2017). Water fluoridation in Brazilian cities at the first decade of the 21st century. Rev Saude Publica.

[2] Watt RG, Daly B, Allison P, Macpherson LM, Venturelli R, Listl S (2019). Ending the neglect of global oral health: time for radical action. Lancet.

[3] O’Mullane DM, Baez RJ, Jones S, Lennon MA, Petersen PE, RuggGunn AJ (2016). Fluoride and oral health. Community Dent Health.

[4] Aurélio Peres M, Simara Fernandes L, Glazer Peres K (2004). Inequality of water fluoridation in Southern Brazil--the inverse equity hypothesis revisited. Soc Sci Med.

[5] Silva FB, Frazão P (2018). Sanitation utilities and fluoridation of water supply systems: an ecological study in Brazilian municipalities, 2008-2010. Epidemiol Serv Saude.

[6] Antunes JL, Narvai PC (2010). Dental health policies in Brazil and their impact on health inequalities. Rev Saude Publica.

[7] Prüss-Ustün A, Wolf J, Corvalán C, Neville T, Bos R, Neira M (2017). Diseases due to unhealthy environments: an updated estimate of the global burden of disease attributable to environmental determinants of health. J Public Health (Oxf).

[8] Prüss A, Kay D, Fewtrell L, Bartram J (2002). Estimating the burden of disease from water, sanitation, and hygiene at a global level. Environ Health Perspect.

[9] Norman G, Pedley S, Takkouche B (2010). Effects of sewerage on diarrhoea and enteric infections: a systematic review and meta-analysis. Lancet Infect Dis.

[10] Tundisi JG (2008). Water resources in the future: problems and solutions. Estud Av.

[11] Prüss-Ustün A, Bartram J, Clasen T, Colford JM, Cumming O, Curtis V (2014). Burden of disease from inadequate water, sanitation and hygiene in low- and middle-income settings: a retrospective analysis of data from 145 countries. Trop Med Int Health.

[12] GBD 2015 Mortality and Causes of Death Collaborators (2016). Global, regional, and national life expectancy, all-cause mortality, and cause-specific mortality for 249 causes of death, 1980-2015: a systematic analysis for the Global Burden of Disease Study 2015. Lancet.

[13] United Nations Transforming our world: the 2030 Agenda for Sustainable Development [cited 2021 May 10]. https://sdgs.un.org/2030agenda.

[14] Adams EA, Boateng GO, Amoyaw JA (2016). Socioeconomic and demographic predictors of potable water and sanitation access in Ghana. Soc Indic Res.

[15] Mesike CG, Mojekwu JN (2012). Environmental determinants of child mortality in Nigeria. J Sustain Dev.

[16] Bishai DM, Cohen R, Alfonso YN, Adam T, Kuruvilla S, Schweitzer J (2016). Factors contributing to maternal and child mortality reductions in 146 low- and middle-income countries between 1990 and 2010. PLoS One.

[17] Vargas I, Mogollón-Pérez AS, Unger JP, da-Silva MR, De Paepe P, Vázquez ML (2015). Regional-based Integrated Healthcare Network policy in Brazil: from formulation to practice. Health Policy Plan.

[18] Simpson SH (2011). Of silos and systems: the issue of regionalizing health care. Can J Hosp Pharm.

[19] Brazilian Institute of Geography and Statistics National Basic Sanitation Survey 2008 [cited 2018 Dec 13]. https://www.ibge.gov.br/estatisticas/multidominio/meio-ambiente/9073-pesquisa-nacional-de-saneamento-basico.html?=&t=o-que-e.

[20] Atlas of Human Development in Brazil (2013). Ranking. http://www.atlasbrasil.org.br/ranking.

[21] Ministério da Saúde (2010). DATASUS: informatics department of Brazilian health system. https://datasus.saude.gov.br/informacoes-de-saude-tabnet/.

[22] Ministério da Economia (2008). Secretariat of the national treasury. https://www.tesourotransparente.gov.br/ckan/dataset.

[23] Ministério da Saúde (2006). Discloses the 2006 health pact: SUS consolidation and approves the operational guidelines of the mentioned pact. https://bvsms.saude.gov.br/bvs/saudelegis/gm/2006/prt0399_22_02_2006.html.

[24] Rezende S, Wajnman S, Carvalho JA, Heller L (2007). Integrating supply and demand of water and sanitation services: hierarchical analysis of the urban Brazil in 2000. Eng Sanit Ambient.

[25] Roncalli AG, Noro LR, Cury JA, Zilbovicius C, Pinheiro HH, Ely HC (2019). Water fluoridation in Brazil: regional distribution and accuracy of information on surveillance in municipalities with more than 50,000 inhabitants. Cad Saude Publica.

[26] Wilson Center (2016). Trajectories of inequality in Brazil. https://www.wilsoncenter.org/event/trajectories-inequality-brazil.

[27] Leoneti AB, do Prado EL, de Oliveira SV (2011). Basic sanitation sector in Brazil: overview about investments and sustainability for the 21st century. Rev Adm Pública.

[28] Novotný J, Hasman J, Lepič M (2018). Contextual factors and motivations affecting rural community sanitation in low- and middle-income countries: a systematic review. Int J Hyg Environ Health.

[29] Luby SP, Rahman M, Arnold BF, Unicomb L, Ashraf S, Winch PJ (2018). Effects of water quality, sanitation, handwashing, and nutritional interventions on diarrhoea and child growth in rural Bangladesh: a cluster randomised controlled trial. Lancet Glob Health.

[30] Petersen PE, Baez RJ, Ogawa H (2020). Global application of oral disease prevention and health promotion as measured 10 years after the 2007 World Health Assembly statement on oral health. Community Dent Oral Epidemiol.

[31] Freire Mdo C, Reis SC, Figueiredo N, Peres KG, Moreira Rda S, Antunes JL (2013). Individual and contextual determinants of dental caries in Brazilian 12-year-olds in 2010. Rev Saude Publica.

[32] Nascimento Sd, Frazão P, Bousquat A, Antunes JL (2013). Dental health in Brazilian adults between 1986 and 2010. Rev Saude Publica.

[33] Freitas CH, Sampaio FC, Roncalli AG, Moysés SJ (2013). Methodological discussion about prevalence of the dental fluorosis on dental health surveys. Rev Saude Publica.

